# ”Predictive Factors and Nomogram for 30-Day Mortality in Heatstroke Patients: A Retrospective Cohort Study”

**DOI:** 10.5811/westjem.48882

**Published:** 2025-11-26

**Authors:** Jeffrey R. Stowell, Geoff Comp, Paul Pugsley, Megan McElhinny, Murtaza Akhter

**Affiliations:** * Creighton University School of Medicine, Phoenix, Department of Emergency Medicine, Phoenix, Arizona; † University of Arizona College of Medicine, Phoenix, Department of Emergency Medicine, Phoenix, Arizona; ‡ Valleywise Health, Department of Emergency Medicine, Phoenix, Arizona; § Penn State Health Milton S. Hershey Medical Center, Department of Emergency Medicine, Hershey, Pennsylvania; || HCA Healthcare, Department of Emergency Medicine, Miami, Florida

**Dear Editor:**

With the continued rise in global environmental temperatures, and the resultant increase in heatstroke deaths,^1^ we would like to recognize and highlight the importance of the article “Predictive Factors and Nomogram for 30-Day Mortality in Heatstroke Patients: A Retrospective Cohort Study,” from Li et al published in May 2025.^2^ We agree that the management of heatstroke continues to be of increasing importance, which the study authors further support with their findings. However, after reviewing their methods, we believe further study participant detail could enhance readers’ understanding and applicability of their findings.

The study authors cite the Chinese Expert Consensus on the Diagnosis and Treatment of Heatstroke to support their study inclusion criteria, which defines heatstroke as “a core temperature of > 40 °C AND abnormalities of the central nervous system, including changes in mental status, convulsions or coma AND accompanied by life threatening multiple organ damage.”^3^ However, the reported study inclusion criteria require only one of the following heatstroke-associated features: central nervous system dysfunction; core body temperature exceeding 40 °C; or functional impairment of multiple organs. According to the study results, the mean core temperature at admission was 39.09 °C. We feel this necessitates further detail of how many of the patients included in the study met the entirety of the heatstroke diagnostic criteria set forth by Liu et al, and specifically how many were noted to be hyperthermic, > 40 °C, at any point during their illness. A further description of the study population, clarifying the number of patients who were hyperthermic at any point, including prehospital temperatures, would help readers better interpret the study cohort.

We would also ask for a further description of the cooling therapy used in this study. The study authors note that “the average cooling time was 3.13 hours.” Given that the average core temperature at admission in the study was 39.09 °C and the stated goal temperature for cooling cessation was 38.5 °C, or approximately a 0.59 °C reduction, this results in an approximate average rate of cooling of 0.003 °C per minute. Comp et al noted that cooling rates can be as fast as 0.13 °C per minute with cold water immersion.^4^ Further, the study authors note that “the average time from onset to initiation of treatment was 8.29 hours.” We concur with their assessment of the importance of initiating heatstroke care within the “golden window” of the first 30 minutes post heat exposure.^5^ The study authors report “a lower risk of death when the core temperature at 30 minutes after admission was below 39.5 °C,” yet it is unclear how often this occurred in this study as the care was, on average, initiated many hours after exposure, and most often lasted over three hours once initiated. How do the study authors believe the delay in the initiation of treatment and the prolonged cooling duration may have impacted their findings? Was this considered when developing the risk predictions? Further clarification of the cooling treatment, including how many patients were treated within the “golden window,” and the outcomes among this group, we believe, would be helpful to better understand the study findings.

Overall, we applaud the study authors for their work and agree that this contributes to our current understanding of heatstroke management, continuing to emphasize the importance of timely cooling therapy. We are optimistic that additional descriptions of the study participants and the therapeutic interventions will further strengthen this work.

We appreciate your consideration of these questions and welcome any feedback. Thank you.

## References

[1] Khatana SAM, Eberly LA, Nathan AS (2023). Projected change in the burden of excess cardiovascular deaths associated with extreme heat by midcentury (2036–2065) in the contiguous United States. Circulation.

[2] Li A, Zhang Y, Zhang X (2025). Predictive factors and nomogram for 30-day mortality in heatstroke patients: a retrospective cohort study. West J Emerg Med.

[3] Yangtai G, Xiaokun Q, Huiyu F (2022). Expert consensus on the diagnosis and treatment of clinically isolated syndrome in China. Chinese J Neurol.

[4] Comp G, Pugsley P, Sklar D (2024). Heat stroke management updates: a description of the development of a novel in-emergency department cold-water immersion protocol and guide for implementation. Ann Emerg Med [Internet].

[5] Heled Y, Rav-Acha M, Shani Y (2004). The “golden hour” for heatstroke treatment. Mil Med.

